# A particle-resolved framework for quantifying microbial colonization and vector risk on environmental microplastics

**DOI:** 10.1016/j.eehl.2026.100245

**Published:** 2026-04-28

**Authors:** Gurusamy Kutralam-Muniasamy, V.C. Shruti

**Affiliations:** a Centro de Investigación e Innovación Tecnológica (CIITEC), Instituto Politécnico Nacional, México 02250, Mexico; b Department of Biotechnology and Bioengineering, Centro de Investigación y de Estudios Avanzados del Instituto Politécnico Nacional, México 07360, Mexico

**Keywords:** Plastisphere, Microbial colonization, Risk assessment, Microplastic vectors, Methodology, Environmental monitoring

## Abstract

Microplastics are widely regarded as vectors for microbial pathogens and antibiotic resistance genes, yet this inference is largely derived from laboratory studies and bulk environmental omics approaches that pool many particles and obscure colonization heterogeneity. Such aggregated analyses demonstrate microbial presence but do not quantify how frequently or unevenly colonization occurs across individual particles, leading to systematic overestimation of universal vector risk. Here, we propose a particle-resolved ecological framework that shifts inference from aggregate detection to population-level quantification of colonization prevalence. By integrating high-throughput imaging with single-particle molecular analyses, this framework enables resolution of right-skewed colonization distributions, in which a minority of “supercarrier” particles are expected to disproportionately contribute to microbial biomass and functional gene loads. To operationalize this shift, we introduce the Colonization Prevalence Index (CPI), a quantitative metric that measures the proportion of environmental microplastics that exceed empirically defined colonization thresholds. CPI anchors plastisphere research in statistical prevalence rather than cumulative signal strength, allowing colonization to be interpreted as an ecological probability rather than an assumed universal trait. Together, the particle-resolved framework and CPI provide a practical, scalable pathway for linking particle properties, microbial colonization patterns, and vector potential, enabling probabilistic risk assessment and more targeted mitigation strategies. By emphasizing ecological resolution over bulk averages, this approach reframes microplastic-associated microbial risk as a measurable population property that can be empirically tested across environments.

## Highlights

• Defines the “vector illusion” as a key methodological bias in microplastic research.
• Proposes a particle-resolved ecological framework replacing bulk inference.
• Introduces the Colonization Prevalence Index (CPI) for quantifying vector risk.
• Conceptualizes “supercarriers” as a minority driving disproportionate microbial load.
• Reframes microplastic risk from assumed ubiquity to measurable ecological probability.

## Graphical abstract

**Figure:**
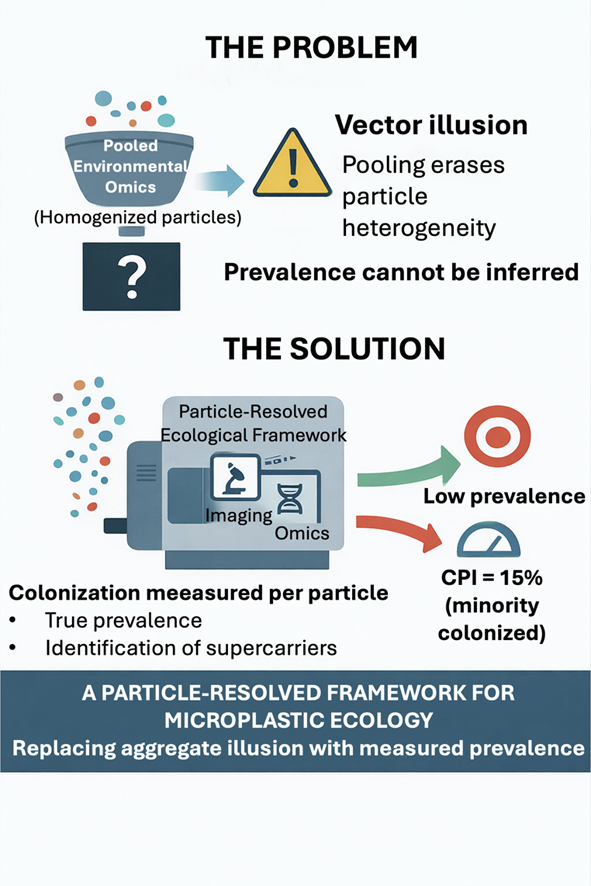

## 1 Introduction

### 1.1 The ubiquitous vector narrative

Microplastics are now detected across ecosystems, ranging from polar ice to human tissues [[1], [2], [3]]. Beyond their ubiquity, they are widely described as vectors for chemical pollutants and microbial agents, including pathogens, viruses, and antibiotic resistance genes (ARGs) [[4], [5], [6]]. Central to this narrative is the plastisphere: microbial communities that colonize plastic surfaces, modify local physicochemical conditions, and facilitate microbial succession and horizontal gene transfer [2,[6], [7], [8]].

The existence of plastisphere communities has reinforced the perception of microplastics as biologically active vectors [9]. However, the ecological scope of these interactions remains poorly constrained. Plastisphere assemblages are highly heterogeneous, shaped by polymer type, environmental aging, and surrounding conditions [10]. Critically, it remains unresolved whether microbial colonization is a widespread, low-intensity phenomenon or instead concentrated within a small subset of particles. This uncertainty limits interpretation of vector relevance and ecological risk.

### 1.2 Methodological pillars and the vector illusion

Claims regarding microplastics as microbial vectors rest primarily on two methodological pillars: controlled laboratory experiments and bulk environmental omics analyses. Laboratory and mesocosm studies demonstrate that microbial colonization can occur under idealized conditions [11,12], while bulk omics approaches pool dozens to hundreds of environmental particles to characterize associated microbial diversity [2,13].

**Fig. 1:**
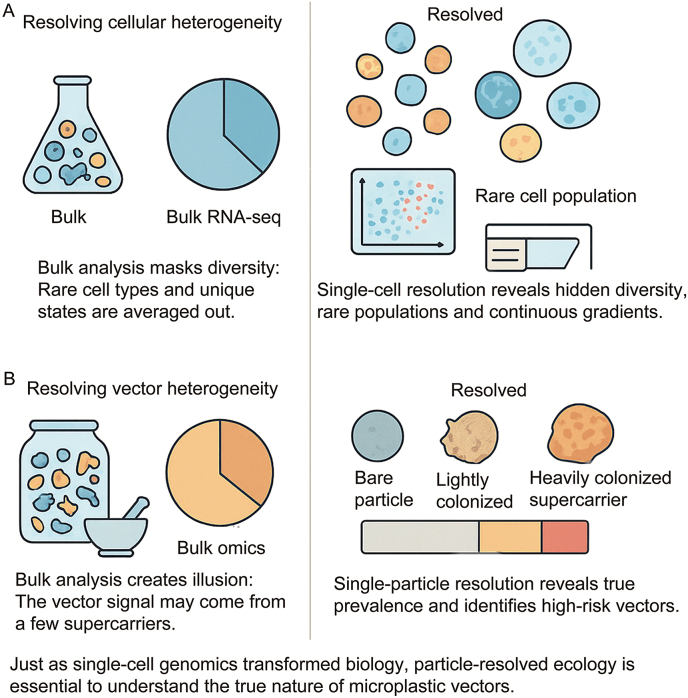
From single cells to single particles: the resolution revolution in ecology. Bulk analyses in both biology and environmental science conflate population averages with universal behavior. The shift from bulk RNA-seq to single-cell omics revealed hidden cellular heterogeneity; an analogous shift to particle-resolved ecology exposes the uneven distribution of microbial colonization among environmental microplastics. This parallel underscores how increasing analytical resolution transforms understanding—from potential to prevalence, from aggregate illusion to ecological reality.

Although these approaches establish mechanistic feasibility, they obscure particle-level heterogeneity. In pooled analyses, cumulative molecular signals may be driven by a small number of heavily colonized “supercarriers,” while the majority of particles remain weakly colonized or uncolonized [10,14]. This aggregation produces what we term a vector illusion, in which bulk signals are misinterpreted as evidence of uniform or pervasive colonization, conflating biological possibility with ecological prevalence [15]. Without particle-resolved inference, it is impossible to distinguish common conditions from rare but intense colonization events (Fig. 1).

### 1.3 From bulk to resolution: Lessons from single-cell biology

Similar inferential challenges have been well documented in other biological fields. In single-cell biology, population-averaged assays long masked critical heterogeneity until single-cell approaches revealed discrete subpopulations with disproportionate functional importance [[16], [17], [18]]. Microplastic research currently occupies an analogous “bulk era”, in which aggregation masks the true distribution of ecological interactions across particles (Fig. 1) [14].

Across disciplines, transitions toward unit-resolved analysis—from single cells to individual mineral grains—have fundamentally reshaped interpretation of ecological and geochemical processes [19,20]. Applying this logic to microplastics suggests that particle-resolved analysis is essential for understanding how colonization is distributed, rather than assuming homogeneity based on pooled signals.

### 1.4 Framing the analytical pivot: A particle-resolved framework

Assessing microplastics as microbial vectors requires a shift from bulk detection to particle-level prevalence. Here, we propose a particle-resolved ecological framework formalized through the Colonization Prevalence Index (CPI), which quantifies colonization as a population-level distribution across individual particles rather than as a bulk average. Despite extensive research on microplastic-associated microbes, systematic quantification of colonization at the single-particle level remains largely absent.

This conceptual and analytical gap limits ecological interpretation and risk assessment. By synthesizing representative studies from microplastic ecology, environmental microbiology, and statistical ecology, we identify how supercarrier-driven signals and methodological aggregation bias current interpretations. This article is not a systematic review; rather, literature was selected through iterative searches in Scopus and Web of Science using core terms (e.g., “plastisphere”, “microbial colonization”, “single-particle analysis”, “antibiotic resistance genes microplastics”) to ensure representative coverage of conceptual and methodological advances.

Sections 2, 3, 4, 5 examine the origins of the vector illusion, principles of particle-resolved ecology, key data gaps, and the CPI framework. Sections 6, 7 discuss methodological implications and future data requirements. To maintain analytical focus, we illustrate CPI using a concise hypothetical example and outline a stepwise workflow for particle-resolved analysis, demonstrating how prevalence-based inference can be generated and interpreted in practice. By introducing a statistically grounded prevalence-based framework, we aim to move beyond assumptions of uniform hazard toward quantitative estimates of ecological relevance within the plastisphere.

## 2 The hidden structure of the plastisphere: Evidence for a skewed colonization landscape

**Fig. 2:**
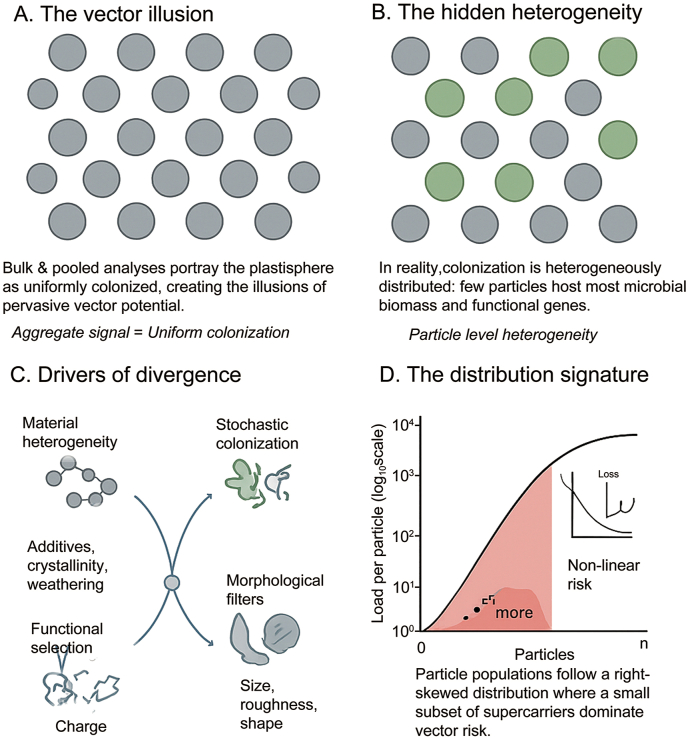
The hidden structure of the plastisphere: From uniform illusion to skewed reality. Bulk analyses create a *vector illusion* of uniform colonization (A), whereas colonization is heterogeneously distributed across particles (B). This divergence arises from material variability, stochastic colonization, and morphological filtering (C), producing a right-skewed distribution where few “supercarriers” dominate vector risk (D).

The widespread characterization of microplastics as universal microbial vectors arises largely from analytical approaches that treat colonization as a uniform, mass-dependent property. While controlled experiments and bulk molecular analyses are mechanistically informative, neither resolves heterogeneity at the scale of individual particles (Fig. 2). As a result, cumulative signals are routinely interpreted as evidence of pervasive colonization, producing an aggregation fallacy in which ecological prevalence is inferred from pooled potential.

### 2.1 Evidence for supercarrier-dominated colonization

The hypothesis that a minority of microplastic particles disproportionately host microbial biomass, pathogens, and mobile genetic elements is consistent with both ecological theory and emerging empirical observations. Uniform colonization is unlikely in systems governed by heterogeneous surfaces and stochastic encounters. Instead, colonization intensity is expected to follow right-skewed distributions characteristic of over-dispersed ecological systems, as described by Taylor’s Power Law [21,22] and the Pareto Principle [23,24].

**Fig. 3:**
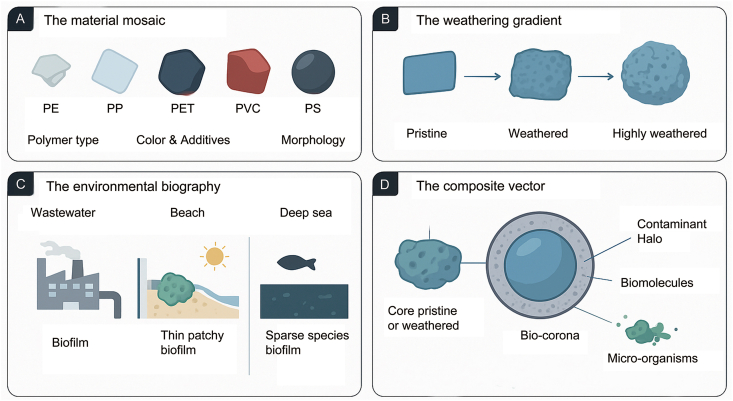
The drivers of microplastic heterogeneity: Why no two particles are alike. Microplastics differ by material composition, weathering state, and environmental history, creating unique colonization niches. (A) Material mosaic—polymer type and additives; (B) Weathering gradient—surface aging; (C) Environmental biography—habitat-specific biofilms; (D) Composite vector—polymer core, biofilm, and contaminant halo.

Multiple abiotic filters generate the variance required for such skew. Even within a single polymer class, manufacturing heterogeneity in additive composition and crystallinity produces diverse surface energies [25,26]. Environmental weathering further amplifies this diversity through oxidation, cracking, and increased roughness, generating microhabitats that alter microbial adhesion probabilities (Fig. 3) [[27], [28], [29]]. Field comparisons show that weathered particles frequently harbor microbial loads distinct from pristine plastics and surrounding matrices [[30], [31], [32], [33]], indicating that surface history often outweighs polymer identity.

Stochastic and biological processes further reinforce heterogeneity. Microbial attachment depends on transient nutrient availability, encounter rates, hydrodynamics, and biogenic particle interactions [10,[34], [35], [36]], allowing initially similar particles to diverge rapidly in colonization state. In parallel, plastisphere metagenomes reveal selective enrichment of ARGs and virulence factors on specific polymers and under specific environmental contexts [[37], [38], [39], [40]], implying that only a subset of particles provides niches conducive to horizontal gene transfer [[41], [42], [43], [44], [45]]. Together, these abiotic, stochastic, and biological filters predict a colonization landscape dominated by a minority of high-impact particles rather than uniform coverage.

### 2.2 Experimental and bulk foundations: Mechanistic strength, distributional blindness

Laboratory and mesocosm studies remain essential for elucidating mechanisms of biofilm formation and polymer–microbe interactions under controlled conditions [11,12]. However, such systems favor colonization success by employing pristine materials, simplified communities, and stable environments, while excluding abrasion, grazing, and turbulence [46]. These conditions inflate colonization rates relative to natural systems [10].

Bulk environmental omics workflows introduce a complementary limitation. By pooling tens to hundreds of particles into a single extract [[47], [48], [49]], they generate molecular profiles representing the mean of an artificially homogenized population. While effective for discovering associated taxa, genes, and viruses [50,51], these approaches conflate presence with prevalence. Detection of a pathogen or ARG may reflect a single heavily colonized particle or diffuse, low-level occurrence across many particles—scenarios with fundamentally different ecological and health implications.

Pooling also severs causal links between microbial load and particle traits such as surface oxidation, roughness, or morphology [37]. Thus, both experimental and bulk approaches excel at identifying what can occur, but are poorly suited to determining how often it occurs or how unevenly it is distributed across particles.

### 2.3 Nonlinear risk and the limits of aggregated inference

When colonization is concentrated within a minority of particles, ecological and toxicological risk becomes inherently non-linear. An environment in which 10% of particles carry the majority of microbial biomass, pathogens, or ARGs is fundamentally different from one in which colonization is uniformly distributed, even if bulk signals are similar [15,[52], [53], [54]]. In such systems, risk depends on the variance and skew of the colonization distribution rather than its mean [21].

By collapsing particle-level diversity into cumulative signals, bulk analyses obscure this structure and implicitly assume linear scaling of risk with particle abundance. Resolving this mismatch requires analytical frameworks that quantify colonization as a probability distribution across individual particles rather than as an aggregate average. This need provides the scientific basis for a particle-resolved ecological framework.

### 2.4 Implications for monitoring and management

These methodological contrasts and their implications for inference are summarized in Table 1. When colonization is concentrated within a minority of particles, bulk approaches may overestimate system-wide risk while obscuring high-impact subsets. Particle-resolved analysis enables monitoring strategies that prioritize particles with traits associated with elevated colonization potential, such as advanced weathering or wastewater-derived biofilms [55,56]. These implications are considered further after the CPI framework is defined and illustrated.

**Table 1:** Paradigm shift in methodological frameworks for assessing microplastic vector risk. Comparison of bulk environmental omics, controlled studies, and the proposed particle-resolved ecological framework.

Aspect	Bulk environmental omics	Controlled studies	Particle-resolved ecology (Proposed)
Core question	Is microbial/viral/ARG signal present in the sample?	*Can* colonization or gene transfer occur?	*How often* are individual particles colonized, and by what?
Unit of analysis	Pooled population (homogenate)	Single particle type/batch under idealized conditions	Individual environmental particle (the composite complex)
Primary output	Cumulative molecular signal (presence/abundance)	Binary or averaged outcome (yes/no; high/low)	Statistical distribution (e.g., CPI)
Handling of heterogeneity	Erased by homogenization	Minimized by experimental design	Quantified as a core property (identifies “supercarriers”)
Link to particle properties	Severed; correlation is impossible	Controlled for a single variable (e.g., polymer)	Directly measured for each particle (enables causal inference)
Assessment of vector risk	“Vector Illusion”: Overestimates prevalence and universality.	“Proof-of-Concept”: Defines hazard but not probability.	“Targeted Risk”: Quantifies prevalence and identifies drivers.
Utility for policy	Screening: Indicates a potential problem exists.	Mechanism: Informs material design and basic science.	Diagnosis & targeting: Guides precise, cost-effective mitigation.

## 3 The imperative for a particle-resolved ecology

The preceding sections reveal a mismatch between how microplastic vector risk is conceptualized and how it is measured. The challenge is no longer to demonstrate that microbes can colonize plastics, but to quantify how colonization is distributed across individual particles and which traits govern that distribution. Addressing this gap requires treating each microplastic particle as a discrete ecological unit rather than as an interchangeable component of a pooled sample.

A particle-resolved ecological framework reframes the central research question from detection to distribution: what proportion of environmental microplastic particles occupy functionally relevant vector states? This shift moves inference from mechanistic possibility to ecological probability, establishing vector risk as a population property rather than an assumed universal trait.

### 3.1 From potential to prevalence: Establishing environmental significance

Single-particle analysis enables the transition from qualitative demonstration to quantitative ecology. Rather than asking whether colonization occurs, it measures how frequently it occurs and under which particle-level conditions across environmental populations. Environmental significance is thus defined by statistical prevalence rather than cumulative signal strength, anchoring inference in empirical distribution data rather than pooled averages [19].

### 3.2 Distribution, nonlinearity, and the basis of vector risk

Bulk analyses implicitly assume that microbial load scales linearly with particle abundance, an assumption violated when colonization follows right-skewed distributions [15]. In such systems, a minority of particles can host most of the microbial biomass, pathogens, or genetic material [[57], [58], [59]], producing fundamentally different risk landscapes despite similar aggregate signals.

Particle-resolved analysis exposes this structure by expressing colonization as a probability distribution across particles rather than as a mass-weighted mean. This distinction allows systems dominated by many lightly colonized particles to be differentiated from those driven by a small number of high-impact “supercarriers”. Formalizing this distributional perspective provides the statistical foundation for the CPI, introduced in Section 5.4.

### 3.3 Linking particle traits to ecological function

Resolving individual particles restores causal linkages between physicochemical traits—polymer type, surface oxidation, weathering state, texture, or additive content—and microbial load. Such associations enable hypothesis testing at the appropriate ecological scale, for example whether specific classes of weathered polymers consistently host denser or more pathogenic biofilms. Trait–function relationships derived from particle-resolved data support predictive ecology and inform targeted mitigation strategies.

### 3.4 Spatial reconstruction of the plastisphere

Microbial ecology is inherently spatial: interactions such as horizontal gene transfer and virus–host exchange occur on discrete surfaces, not within pooled extracts [[60], [61], [62]]. Particle-resolved analysis reconstructs this spatial organization, revealing the plastisphere as a mosaic of microscale ecosystems rather than a homogeneous microbial film. Restoring spatial and population context transforms the plastisphere from a descriptive metaphor into a measurable ecological system.

### 3.5 Scope and practical considerations

Common critiques of particle-resolved approaches emphasize throughput and scalability. However, bulk methods provide screening rather than diagnosis: they indicate that colonization may occur but cannot quantify how prevalent it is. Advances in high-content imaging, flow cytometry, and automated sorting already permit analysis of large particle numbers [[63], [64], [65], [66]], making particle-level inference increasingly feasible. The central issue is not analytical speed, but whether measurements capture ecological reality.

In sum, a particle-resolved framework redefines the ecological unit of inference and provides the conceptual basis for prevalence-based risk assessment. By replacing aggregate inference with statistically resolved distributions, it establishes the foundation for quantitative metrics capable of distinguishing rare, high-impact events from widespread low-level associations. The following section identifies the critical data gaps that must be addressed to operationalize this framework. Table 2 summarizes these gaps, linking common assumptions to the particle-resolved metrics required to test them.

**Table 2:** Defining the critical knowledge gaps and required particle-resolved metrics. Comparative summary of the major unresolved questions in microplastic vector research, the flawed assumptions sustaining them, and the proposed quantitative metrics and expected advances enabled by particle-resolved ecology.

Critical knowledge gap	Current (flawed) assumption	Required particle-resolved metric	Expected impact of new data
Prevalence and Distribution Gap	Colonization is widespread, uniform, and risk scales linearly with particle count.	CPI: The percentage of particles in a population that are substantially colonized. Frequency distribution of colonization intensity: A histogram showing the spread of total microbial and viral load from bare to supercarrier.	Transform risk models from assuming a ubiquitous hazard to modeling the probability of encounter with a supercarrier, based on empirical, non-linear distributions.
Functional Gene Vector Gap	The plastisphere is a uniform, high-risk reservoir for mobile genetic elements like ARGs.	Distribution of ARG/metal resistance gene (MRG) abundance: The frequency and concentration of resistance genes per particle, and their co-localization with viral sequences (inducing transduction potential).	Determine if microplastics are significant vectors for horizontal gene transfer or if this risk is confined to a rare subset of genetic supercarriers, drastically refining the scope of the AMR threat.
Multi-Kingdom Interaction Gap	The primary vector risk is from pathogenic bacteria.	Viral Colonization Prevalence (VCP): The percentage of particles acting as viral hotspots. Co-occurrence network analysis: Mapping the specific associations between bacterial hosts, viruses, and ARGs on individual particles.	Unveil the hidden role of the plastisphere as a platform for virus-host interactions and transduction, identifying a previously invisible dimension of ecological and health risk.
Driver & Predictive Gap	All polymers/particles have similar vector potential.	Trait-colonization correlation matrix: A dataset linking CPI, ARG load, and VCP to specific particle traits (polymer, weathering state, surface topography, and origin).	Enable predictive risk assessment and smart regulation, allowing policymakers and industry to target the specific sources and types of particles that genuinely function as supercarriers.
Composite Vector Gap	The plastic polymer is the relevant ecological unit.	Quantification of the composite particle complex: Metrics defining how the acquired eco-corona (mineral/organic coatings) modifies colonization, stability, and vector function compared to the pristine polymer.	Reframe risk assessment and policy to account for the environmentally transformed particle, leading to more accurate fate, transport, and exposure models.
Risk Interpretation Gap	Detection of colonization equates to significant hazard.	Risk-categorized CPI: Prevalence data linked to proposed risk tiers (Low: CPI <10%; Moderate: 10%–50%; High: >50%). Exposure & trophic transfer coefficients: Metrics linking CPI to particle ingestion rates, gut retention, and tissue translocation in model organisms.	Enables proportionate risk assessment. Moves policy from blanket precaution to evidence-based, tiered action focused on high-CPI environments and particle types.

## 4 The critical knowledge gap: Defining the missing data

Despite extensive research on plastisphere composition, function, and gene content, microplastic ecology lacks a baseline metric describing how microbial colonization is distributed across individual environmental particles. As a result, even the most basic ecological questions remain unresolved: how many particles are colonized, and how unevenly is colonization distributed within environmental populations? This gap has three interrelated dimensions:

(1) Prevalence. What proportion of environmental microplastics within a given habitat are uncolonized, lightly colonized, or densely colonized? At present, no empirical baseline exists for what constitutes typical colonization under natural conditions.

(2) Distribution. Is vector capacity broadly shared among particles or concentrated within a small subset of high-impact “supercarriers”? These contrasting distributions imply fundamentally different ecological dynamics and exposure risks.

(3) Predictability. Which environmental factors (e.g., salinity, nutrient availability, hydrodynamics) and particle traits (e.g., polymer type, surface roughness, weathering state) determine a particle’s probability of becoming a heavily colonized vector?

The absence of particle-resolved data perpetuates inferential uncertainty. Without quantifying prevalence and distribution, laboratory-derived mechanisms cannot be reliably scaled to environmental systems, and risk assessments implicitly assume uniform hazard. Addressing this gap by measuring colonization as a population-level probability rather than an assumed constant is therefore essential.

Resolving these unknowns defines the analytical agenda that follows: to operationalize particle-resolved ecology through quantitative metrics capable of capturing prevalence, distribution, and trait dependence across environmental microplastic populations.

## 5 A proposed framework: Implementing particle-resolved ecology

This section outlines a particle-resolved framework for quantifying microbial colonization across environmental microplastic populations. Colonization is treated as a distribution across individual particles rather than as a pooled signal, enabling direct estimation of prevalence, variance, and trait dependence. Most existing studies rely on laboratory experiments or in situ deployments to resolve mechanisms under controlled conditions [13,17], while comparatively few analyze environmentally isolated particles at sufficient resolution to characterize natural colonization patterns. The framework presented here integrates field-collected particles, standardized in situ exposures, and laboratory-controlled systems within a unified analytical structure.

### 5.1 The tripartite architecture of particle-resolved ecology

The particle-resolved ecological framework is organized around three complementary analytical domains that together span observation, inference, and validation:

Domain 1: Field-collected particles. Environmental particles establish the empirical baseline by quantifying the natural prevalence and variability of microbial colonization under real-world conditions.

Domain 2: In situ processed particles. Standardized materials deployed in authentic environments link observation to causation, allowing environmental drivers of colonization to be assessed while maintaining field relevance.

Domain 3: Laboratory-controlled systems. Controlled experiments test mechanistic hypotheses derived from field and in situ observations, providing reproducible validation of particle-level processes.

Together, these domains form a closed analytical loop in which ecological realism constrains mechanistic interpretation, and experimental precision informs population-level inference. The operational characteristics, analytical resolution, and inferential strengths of each domain are summarized in Table 3 [50,[67], [68], [69], [70], [71], [72], [73], [74], [75], [76], [77], [78], [79]].

**Table 3:** Analytical domains, methodological tiers, and their roles in the particle-resolved ecological framework [50,[67], [68], [69], [70], [71], [72], [73], [74], [75], [76], [77], [78], [79]].

Domain/sampling source	Analytical tier/method	Resolution	Principle/technique	Key outputs	Advantages	Limitations/challenges	Role in CPI framework
Domain 1: Field-collected particles	Bulk extraction & size fractionation	Macro–micro (50 μm–mm)	Gentle manual collection, sieving, or low-density filtration stratified by size and polymer	Particle inventory; population-level sampling units	Preserves natural variability and in situ colonization; high ecological realism	Fragile or adhesive “supercarriers” may be lost; risk of cross-contamination	Defines empirical baseline for CPI (Environmental CPI)
	High-throughput imaging (Pathway A)	Morphological (μm)	Fluorescence/confocal microscopy with ML segmentation (U-Net, CellProfiler)	Biofilm coverage; colonization extent; CPI estimate	Rapid quantification of thousands of particles; scalable	Biomass overestimation from debris or stains; limited functional data	Primary CPI estimator for colonization prevalence and distribution
	Spectroscopic correlation (μFT-IR/Raman/XPS)	Chemical/surface	Optical and surface spectroscopy	Polymer identity, oxidation, coating composition	Establishes abiotic context and substrate heterogeneity	Minimal biological information; time-intensive	Links polymer and surface traits to colonization patterns
Domain 2: In situ processed particles	Controlled environmental exposure	Field realism + experimental control	Deployment of known materials (substrate panels, resin beads, films) for natural colonization	Colonization rate, succession, and environmental modulation	Directly links colonization to habitat conditions and substrate properties	Retrieval logistics; exposure duration bias	Quantifies colonization dynamics; validates CPI drivers
	Imaging flow cytometry (IFC)	Morphological/fluorescence	Flow-based imaging (e.g., ImageStream®)	Size, shape, fluorescence intensity	Automated classification; high throughput	Reduced spatial resolution; requires calibration	Rapid screening for CPI estimation across exposure series
	Correlative imaging (CARD-FISH, Cryo-CLEM, MALDI-MSI, NanoSIMS)	Structural + molecular (nm–μm)	Spatial co-localization of taxa, metabolites, or functional genes	Microbial localization; ARG or metabolite mapping	Integrates structure, taxonomy, and function; high specificity	Low throughput; advanced instrumentation	Links morphology, taxonomy, and functional traits on individual particles
Domain 3: Laboratory-controlled systems	Single-particle omics (Pathway B)	Taxonomic/functional	Low-biomass WGA, 16S or shotgun metagenomics, Nanopore long-read sequencing	Taxonomic composition, ARGs, virulence and metabolic genes	Direct causal link between particle traits and microbial load	Contamination risk; distinguishing attachment vs. proximity	Provides functional layer of CPI (biological intensity component)
	Functional assays (Metatranscriptomics, Microfluidic biosensing)	Activity/gene expression	RNA-seq, optical or enzymatic biosensing	Active metabolism, stress, virulence expression	Captures real-time functional activity	High RNA demand; transient expression; costly	Defines functional CPI (CPIf); adds dynamic activity layer
	Microarray/microfluidic chip platforms	Parallel, high-content	Chip-based imaging + spectroscopy	Multi-parameter analysis of hundreds of particles	Automatable, multiplexed, scalable	Requires signal correction and calibration	Enables industrial-scale CPI and inter-lab harmonization
Cross-domain validation	Automation & machine learning integration	Data-fusion scale	AI-driven segmentation and model fusion	Automated CPI computation; “supercarrier” prediction	Improves reproducibility and comparability	Needs open datasets and benchmark models	Core enabler of high-throughput, standardized CPI pipelines
	Spike-recovery & mock community experiments	Calibration scale	Defined biofilm consortia on synthetic particles	Recovery efficiency, detection limits, threshold bias	Quantitative validation of CPI precision and bias correction	Labor-intensive; requires standardized reference materials	Establishes analytical confidence and cross-lab calibration

### 5.2 Sampling for particle-resolved ecology

Accurate estimation of colonization prevalence depends fundamentally on how particles are collected. Sampling must be stratified across particle size fractions and environmental matrices to ensure representative coverage of both common and rare particle types. Because chemical digestion and aggressive extraction disrupt biofilms, particle-resolved ecology prioritizes recovery methods that preserve microbial and extracellular structures.

Across matrices, manual isolation remains the most reliable means of maintaining ecological fidelity. In sediments and soils, particles are recovered through sieving followed by stereomicroscopic selection [62]. In biotic samples, dissection and direct isolation of embedded particles preserves spatial context and enables particle-specific microbial analysis [49]. In aquatic systems, filtration through inert meshes or membranes followed by sterile manual selection allows individual particles to be recovered without cross-contamination [80].

A critical limitation of manual recovery is operator selection bias. Visual selection preferentially captures larger or more conspicuous particles, potentially under-representing small, transparent, or heavily biofilm-covered fragments that may differ systematically in colonization potential [80]. Without explicit attention to this bias, prevalence estimates may be skewed and the composition of high-impact “supercarrier” subsets mischaracterized.

In situ incubations provide a complementary strategy. Pre-cleaned polymer fragments or films deployed in natural environments undergo colonization under authentic conditions and are analyzed using the same single-particle workflows as field-collected specimens [10,12]. Laboratory exposure experiments follow analogous protocols under controlled conditions, enabling direct comparison of colonization kinetics across environmental and experimental domains [11].

Importantly, most existing extraction and density-separation workflows were designed for polymer recovery rather than ecological preservation [81,82]. High-density solutions, oxidants, and surfactants commonly used for chemical identification can strip away the microbial structures central to particle-resolved inference [13]. The particle-resolved framework therefore reframes sampling as an act of ecological preservation rather than purification, emphasizing gentle retrieval, minimal processing, and documentation of particle context to ensure that downstream analyses reflect true environmental colonization.

### 5.3 Operationalizing the particle-resolved ecological framework

**Fig. 4:**
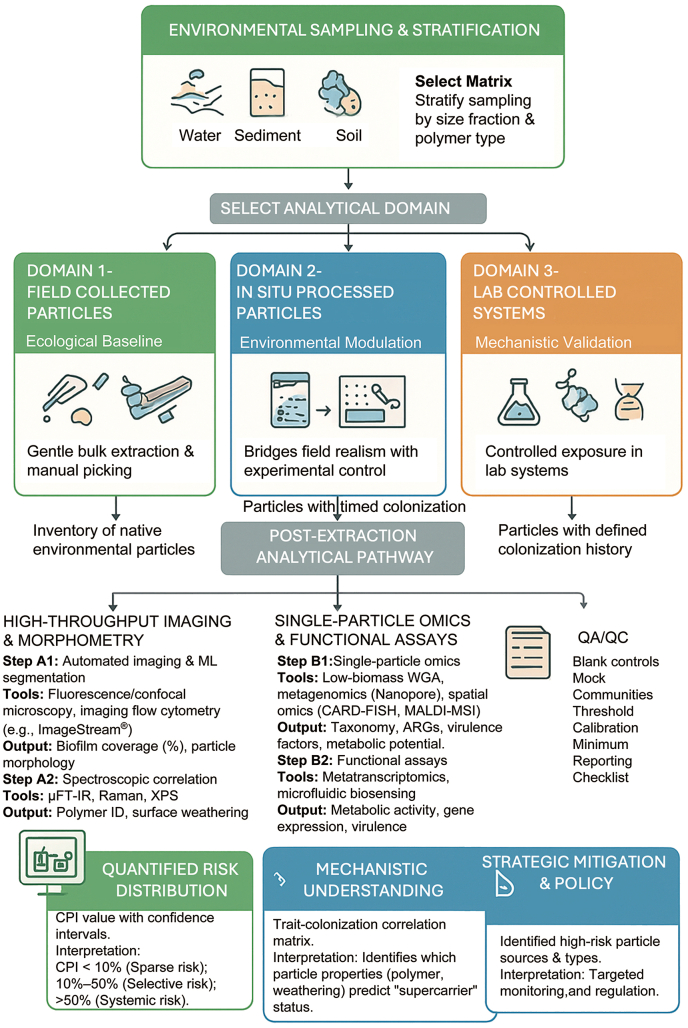
From Extraction to Ecological Inference: Decision Tree for Implementing Particle-Resolved Ecology. The framework links sampling and analysis across three domains—field-collected, in situ processed, and laboratory-controlled particles—each contributing to CPI development. Imaging-based (Pathway A) and molecular analyses are integrated to quantify colonization prevalence and intensity, with standardized QA/QC ensuring reproducibility. Together, these workflows generate particle-resolved, ecologically grounded evidence for vector risk assessment.

Particle-resolved ecology begins where conventional workflows typically end—after particle recovery but before homogenization. Instead of pooling material, each microplastic is treated as a discrete ecological unit. Implementation proceeds through two complementary analytical routes—optical and molecular—that together enable population-level inference (Fig. 4).

The first route, high-throughput imaging, provides rapid quantification of colonization extent and spatial organization. Fluorescence and confocal microscopy, combined with machine-learning segmentation, can distinguish microbial biomass and extracellular polymeric substances from mineral or organic debris, yielding quantitative estimates of surface coverage per particle [50,68]. Imaging flow cytometry and automated high-content microscopy extend this approach to thousands of particles per run, enabling statistically robust estimates of colonization prevalence across environmental populations [67].

The second route, single-particle omics, resolves taxonomic and functional attributes by sequencing material directly associated with individual particles. Low-biomass molecular workflows—including whole-genome amplification, nanopore sequencing, and spatially resolved methods such as CARD-FISH or mass spectrometry imaging—allow characterization of microbial diversity and gene content while preserving particle-specific context [69,71,83].

These routes can be integrated through microfluidic and microarray platforms that combine imaging, polymer identification, and molecular interrogation within unified workflows [72,73]. Automated sorting and fluorescence-activated particle isolation enable particles identified during imaging as potential “supercarriers” to be selectively routed to molecular assays, improving efficiency and interpretability [70,74]. Together, these adaptations transform single-particle analysis from a manual, low-throughput exercise into a scalable, semi-automated pipeline capable of resolving colonization distributions within realistic sampling efforts [69,76]. Importantly, this framework relies on adapting existing technologies from microbiology, cytometry, and materials science rather than developing entirely new tools [84,85].

Several technical limitations remain. Gentle extraction may underestimate fragile biofilms or loosely attached aggregates [86]. Fluorescent staining cannot always distinguish intact cells from extracellular debris, and mineral coatings may generate false positives [87,88]. Molecular approaches face challenges related to contamination, amplification bias, and surface-association ambiguity [77,78]. Integrating imaging, spectroscopic, and sequencing data requires harmonized metadata and rigorous cross-calibration, while machine-learning segmentation depends on diverse, well-annotated training datasets to avoid systematic misclassification [75,79]. These constraints are technical rather than conceptual and are addressable through standardized protocols and transparent reporting [69].

By combining large-scale imaging with targeted molecular validation, the particle-resolved framework yields distributional data rather than cumulative averages. It quantifies not only whether colonization occurs, but how frequently and with what intensity across natural particle populations. This capability provides the statistical foundation for computing the CPI, which formalizes colonization as a measurable ecological probability rather than an inferred bulk property. The following section details CPI formulation, calibration, and interpretation as the core quantitative output of the framework.

### 5.4 Synthesizing the tripartite data: CPI

CPI is the core quantitative output of the particle-resolved ecological framework, translating single-particle observations into population-level inference (Text S1). CPI quantifies the proportion of particles within an environmental sample that are substantially colonized, capturing ecological prevalence rather than cumulative potential [50,68].

Formally,

*CPI* = (*N*_*c*_/*N*_*t*_) × 100%

Where, *N_c_* is the number of colonized particles; *N_t_* is the total number analyzed. Crucially, *CPI* depends on an operational, data-driven definition of “colonized,” which must be explicitly justified rather than assumed.

1) Defining colonization thresholds

Colonization status is determined using objective thresholds tailored to the analytical pathway:

Pathway A (Imaging): A particle is considered colonized when its biofilm coverage exceeds a defined threshold (Tb). Tb should be determined via receiver operating characteristic (ROC) curve analysis by comparing automated segmentation results against a manually curated validation set, as demonstrated in biofilm quantification studies [68,89]. The threshold that maximizes the Youden Index (sensitivity + specificity – 1) provides an objective balance between true colonization and false positives from debris or staining artifacts.

Pathway B (Omics): Colonization is established when microbial load (e.g., 16S rRNA gene copies or read counts) exceeds a threshold (Tm). Tm should be derived from blank distribution analysis, ideally set as the mean plus three standard deviations of signals from negative controls (e.g., extraction blanks), following established practices for low-biomass molecular workflows [71,83]. This ensures the threshold exceeds 99.7% of background contamination, minimizing false-positive colonization calls.

These approaches ensure CPI values are reproducible, comparable across studies, and robust to analytical variation.

2) Accounting for colonization intensity

To incorporate heterogeneity in colonization strength, a weighted CPI (CPI_w_) can be derived as

*CPI_w_* = (1/*N_t_*) Σ (*I_i_*/*I_max_*)

Where, *I_i_* is the biofilm coverage or microbial load for particle *i*; *I_max_* is the maximum observed. *CPI*_*w*_ thus gives greater weight to heavily colonized “supercarrier” particles and provides a continuous gradient of vector potential. The operational workflow for calculating the CPI—from image analysis and threshold determination to final prevalence estimation—is illustrated in detail through a worked hypothetical example in Text S2.

3) Sampling design and statistical confidence

Reliable CPI estimation requires statistically justified sample sizes rather than empirical rules of thumb. For single-population prevalence estimates, binomial sampling theory indicates that achieving ±5% precision at 95% confidence requires about 140 particles when CPI is 10% and about 385 particles when CPI is 50%. For comparative analyses (e.g., among habitats or polymer types), power analysis for a two-proportion test (α = 0.05, power = 0.80) indicates that detecting a 15-percentage-point difference in CPI requires about 200 particles per group, while detecting a 10-point difference requires about 450 particles per group. These estimates assume independent particles; clustering within samples may reduce effective sample size. Accordingly, we recommend sampling at least 200–300 particles per population or experimental group, stratified across relevant particle categories where applicable, with confidence intervals estimated using appropriate binomial or bootstrap methods [77]. When combining data across sites, hierarchical or mixed-effects models should account for the nested structure (particles → samples → sites).

4) Quality assurance and threshold calibration

Robust estimation of the CPI requires standardized, empirically justified thresholds for defining colonized particles, together with rigorous quality assurance (QA) and contamination control. Colonization thresholds (Tb for imaging; Tm for omics) must be derived from control-derived distributions rather than set arbitrarily, ensuring that particle signals exceed background noise and laboratory contamination. Each analytical batch should include comprehensive negative controls—including reagent blanks, process blanks (empty-pick controls), imaging negatives, and sequencing blanks—with colonization calls accepted only when signals exceed conservative, blank-based cutoffs. CPI sensitivity should be evaluated across plausible threshold ranges (e.g., 10%–30% surface coverage; 50th–90th percentile of blank-corrected read distributions) to demonstrate robustness.

To minimize false positives from abiotic debris, mineral coatings, or auto-fluorescent material, colonization assignments should be validated on a representative subset of particles using orthogonal approaches. These include optical or confocal imaging with nucleic acid and polysaccharide-specific stains, mild enzymatic disruption of biofilms, spatial confirmation of microbial signals on polymer surfaces via μFT-IR or Raman mapping, and high-resolution surface topology assessment with SEM. Particles that cannot be unambiguously classified should be reported as “composite particles” rather than forced into binary categories, enhancing transparency and reproducibility.

Finally, spike-recovery experiments with synthetic particles and mock microbial communities are essential to quantify particle recovery, biofilm loss, amplification bias, and detection limits. These benchmarks allow correction for systematic bias and ensure that CPI estimates are reproducible, quantitatively robust, and comparable across studies, providing a reliable framework for particle-resolved microbial ecology.

5) Data processing, standardization, and transparency

Segmentation pipelines (e.g., U-Net–based architectures) should be trained on annotated image libraries spanning polymers, coatings, and particle sizes; performance metrics (precision, recall, intersection-over-union) must be reported for reproducibility. Deep-learning frameworks such as MP-Net and skip-connected U-Net variants have demonstrated high accuracy for fluorescence-based and hyperspectral segmentation of microplastics across diverse morphologies [[90], [91], [92], [93]].

All raw images, segmentation masks, sequence reads, and CPI scripts should be deposited in open repositories (e.g., Dryad, Zenodo, NCBI SRA) to promote reproducibility and transparency. The need for standardized reporting and metadata completeness has been emphasized in recent data-harmonization initiatives within the microplastics field [94,95]. A Particle-Resolved Ecology Minimum Reporting Checklist (Text S3) ensures comparability across laboratories by requiring standardized metadata: sampling details, recovery efficiency, imaging parameters, thresholds, and statistical methods.

6) Interpretation and integration across domains

CPI values can be interpreted in risk-relevant categories: CPI <10% indicates sparse colonization and localized risk, while CPI >50% suggests systemic vector potential. Importantly, CPI characterizes the composite environmental particle—the plastic core plus its persistent organic and mineral coatings—reflecting ecological function rather than pristine material properties [96].

The CPI unifies data from all three analytical domains, integrating empirical prevalence from field-collected particles, environmental modulation from in situ exposures, and mechanistic validation from laboratory systems. These complementary inputs transform the CPI from a descriptive statistic into a predictive ecological indicator that captures the true heterogeneity of vector potential across particle populations [97].

By anchoring microplastic risk assessment in measured prevalence rather than cumulative inference, the CPI provides a statistically resolved, ecologically grounded perspective on hazard—bridging high-content analytical pipelines with transparent, FAIR-compliant data structures [94,97].

### 5.5 Advantages and limitations of the particle-resolved framework

The particle-resolved ecological framework advances microplastic research by replacing pooled averages with population-level distributions. By treating each particle as a discrete ecological unit, it resolves colonization heterogeneity and distinguishes widespread colonization from concentration within a minority of “supercarriers” [67,69]. This resolution enables computation of the CPI, converting cumulative molecular signals into statistically defined prevalence metrics [50,83]. Linking colonization intensity to material traits—such as polymer type, weathering state, or surface morphology—supports causal inference and yields metrics that are comparable across environments and studies [94,96].

These advantages are accompanied by technical constraints. Single-particle omics operates near detection limits and requires stringent contamination control and validation [77,78]. Manual isolation and analysis remain resource-intensive, highlighting the need for automation and machine-learning–based segmentation to achieve scalability [[91], [92], [93]]. CPI thresholds must therefore be empirically calibrated and interpreted as properties of the composite environmental particle rather than pristine polymer identity [96].

Overall, these limitations are technical and tractable through standardization and automation. In contrast, the framework’s core contribution is conceptual: it enables statistically grounded assessment of colonization prevalence and heterogeneity, providing a more realistic basis for ecological interpretation and risk-relevant inference in plastisphere research [94,95].

## 6 Expected outcomes and novel insights

Implementing a particle-resolved ecological framework redefines how the plastisphere is measured and understood. It replaces the reliance on pooled inference with statistically resolved data, advancing microplastic research toward quantitative ecology.

### 6.1 Quantifying prevalence and redefining scale

The first application of the CPI will yield population-level distributions of microbial colonization across environmental microplastics. These data will establish whether the plastisphere represents a widespread ecological condition or a rare, localized phenomenon—replacing the presumption of ubiquity with measurable prevalence and recalibrating the scale of vector risk [2].

### 6.2 Identifying high-risk traits and supercarriers

Linking CPI to particle traits such as polymer identity, surface roughness, and weathering will reveal which features predict dense colonization. This evidence enables precision-focused management of high-risk “supercarriers,” guiding monitoring and mitigation toward the minority of particles that dominate biological and chemical vectoring.

### 6.3 Integrating field, laboratory, and modeling realms

Particle-resolved datasets will close the gap between observation and causation. Trait combinations identified in nature can be reproduced under controlled conditions, while CPI values inform probabilistic terms in risk and transport models—establishing a feedback loop between empirical data and predictive ecology.

### 6.4 Extending beyond the plastisphere

The framework’s logic—resolving heterogeneity at the unit of ecological interaction—applies broadly to aerosols, soot, tire wear, and nanomaterials. Microplastics thus serve as the test case for a general theory of particulate ecology, grounding environmental risk assessment in measured distributions rather than aggregated assumptions [98,99].

### 6.5 From CPI to risk: Defining interpretable categories

The CPI enables translation of colonization prevalence into actionable risk tiers. While regulatory thresholds need further validation, we propose: low risk (CPI < 10%) for sparse, localized colonization with minimal impact; moderate risk (CPI 10%–50%) for common but not ubiquitous colonization, indicating ecosystem-level vector potential; and high risk (CPI > 50%) for widespread colonization, signaling high encounter probability and significant ecological or exposure implications. This tiered, prevalence-based framework reframes vector risk from qualitative assumption to quantitative assessment, supporting targeted and proportionate environmental management.

## 7 Future directions and conclusion

### 7.1 Priority research trajectories: From data to dynamics

The particle-resolved framework provides the essential tools to address the critical data gap: large-scale, quantitative colonization intensity distributions across natural microplastic populations. At present, such quantitative data remain scarce, underscoring the need for coordinated, large-scale CPI applications to empirically evaluate the supercarrier hypothesis. Priority research must therefore focus on applying the CPI across diverse ecosystems to test how environmental drivers shape colonization prevalence. Future work should track individual particles over time to reveal assembly, maturation, and dissipation dynamics, and scale spatially from microhabitats to catchments, estuaries, and global gradients. Crucially, integrating CPI measurements with trophic transfer and exposure studies—such as ingestion assays, gut retention analyses, and tissue translocation experiments—will bridge the gap between environmental prevalence and internal biological dose, enabling true probabilistic risk assessment.

### 7.2 Technological and analytical frontiers

Realizing this research agenda requires advances in analytical integration and automation. Combining high-throughput fluorescence imaging (Pathway A) with molecular and spatial mapping (Pathway B) through techniques like CARD-FISH, microarray-based hybridization, or cryo-correlative microscopy will enable simultaneous visualization of morphology, taxonomy, and function within single particles. Automation of particle isolation, imaging, and classification is essential to scale analyses to tens of thousands of particles, facilitating inter-laboratory comparability and the creation of standardized, open CPI databases. Method validation through spike-recovery experiments, mock communities, and longitudinal deployments will benchmark recovery efficiency, detection limits, and the temporal persistence of supercarrier states.

### 7.3 Scientific, technical, and societal implications

Adopting this framework carries significant cross-sector implications. Scientifically, it transitions plastisphere research from qualitative, bulk-level description to a quantitative, predictive ecology grounded in population distributions. Technically, it drives innovation in automated, multi-modal single-particle analysis, creating scalable tools for environmental diagnostics and monitoring. Societally, it enables targeted risk assessment and evidence-based policy, shifting resource allocation from indiscriminate mitigation toward precise intervention focused on genuine high-risk sources, particle types, and environments, thereby enhancing the cost-effectiveness and ecological rationale of management strategies.

### 7.4 Conclusion: From illusion to accountable evidence

The persistent portrayal of microplastics as ubiquitous microbial vectors exemplifies the Aggregation Fallacy—inferring uniform hazard from cumulative signals. Particle-resolved ecology directly challenges this illusion by demanding statistical, distributional evidence of prevalence. This methodological shift restores precision and accountability to the field, transforming an unbounded narrative into a tractable ecological system governed by definable probabilities. By quantifying colonization as a population-level property, the framework redefines what counts as credible evidence in particulate ecology, applying not only to microplastics but to any particulate contaminant (e.g., soot, aerosols, nanomaterials) where aggregate measurements obscure risk. Ultimately, this epistemic transition—from assumption to evidence, and from narrative to nature—anchors environmental science in empirical humility and statistical truth, defining the standard by which future microplastic risk will be measured and mitigated.

## CRediT authorship contribution statement

**Gurusamy Kutralam-Muniasamy:** Writing – review & editing, Writing – original draft, Investigation, Formal analysis, Conceptualization. **V.C. Shruti:** Writing – review & editing, Writing – original draft, Investigation, Funding acquisition, Formal analysis, Conceptualization.

## Declaration of generative AI and AI-assisted technologies in the writing process

During the preparation of this work the author(s) used ChatGPT in order to give suggestions on text in order to improve readability and writing style. After using this tool/service, the author(s) reviewed and edited the content as needed and take(s) full responsibility for the content of the publication.

## Declaration of competing interest

The authors declare that they have no known competing financial interests or personal relationships that could have appeared to influence the work reported in this paper.
