# Supplementary material for: A particle-resolved framework for quantifying microbial colonization and vector risk on environmental microplastics
Source: Eco Environ Health. 2026 Apr 28;5(2):100245. doi: 10.1016/j.eehl.2026.100245 (PMC13213754; doi:10.1016/j.eehl.2026.100245)
Supplement: Multimedia component 1 [file mmc1.docx]

**Supplementary Material**

**for**

**A Particle-Resolved Framework for Quantifying Microbial Colonization and Vector Risk on Environmental Microplastics**

Gurusamy Kutralam-Muniasamy^a,*^, V.C. Shruti^b,*^

^a^ Centro de Investigación e Innovación Tecnológica (CIITEC), Instituto Politécnico Nacional, México 02250, México

^b^ Department of Biotechnology and Bioengineering, Centro de Investigación y de Estudios Avanzados del Instituto Politécnico Nacional, México 07360, México

**^*^**Corresponding authors

Text S1 | Validation and Calibration Experiments for the Particle-Resolved Framework

Text S2 | Worked Example of CPI Calculation and Interpretation

Text S3 | Minimum Reporting Checklist for Particle-Resolved Ecology and CPI Studies

**Number of Texts: 3**

**Number of Pages: 10**

**Number of Supplementary Materials: 3**

**Submitted to Eco-Environment and Health**

**Text S1 | Validation and Calibration Experiments for the Particle-Resolved Framework**

**Overview**

To ensure analytical validity and ecological interpretability of the Colonization Prevalence Index (CPI) and associated particle-resolved datasets, a series of modular validation experiments are recommended. These protocols quantify recovery efficiency, detection bias, and the fidelity of CPI estimates under controlled and semi-natural conditions. Together, they calibrate how laboratory performance scales to environmental complexity.

**1. Spike-Recovery and Detection Sensitivity Experiment**

**Objective:**
Quantify the recovery efficiency and detection limits of the particle-resolved workflow from environmental matrices.

**Rationale:**
Particle recovery during density separation, handling, and imaging is not 100%. Known spikes allow calibration of CPI and weighted CPI (CPI₍w₎) against true colonization frequencies.

**Experimental Steps:**

1. Prepare a batch of sterile polymer fragments (e.g., PE or PET, 100–300 µm) and a set of identical fragments coated with a standardized microbial biofilm (e.g., *Pseudomonas fluorescens* expressing GFP).
2. Spike a known number of colonized (N_c) and uncolonized (N_u) particles into sediment or water matrices at realistic concentrations (10²–10⁴ particles/L or g).
3. Process samples through the standard extraction and imaging workflow (density separation, visual sorting, fluorescence imaging).
4. Quantify the proportion of recovered colonized particles relative to the known spike composition.

**Expected Output:**
Measured CPI_recovered vs. CPI_true.

**Validation Metric:**
Recovery efficiency = (CPI_recovered/CPI_true) × 100%.
Target ≥ 80% consistency across triplicates. Deviations quantify systematic bias in extraction or imaging detection.

**2. Mock-Community Colonization and Omics Validation**

**Objective:**
Benchmark taxonomic and functional accuracy of single-particle omics workflows.

**Rationale:**
Low-biomass sequencing can introduce stochastic dropouts and contamination; defined mock biofilms establish detection thresholds and contamination correction factors.

**Experimental Steps:**

1. Generate mock biofilms on inert beads or plastic fragments using defined bacterial consortia (e.g., *Pseudomonas putida*, *Vibrio parahaemolyticus*, *Bacillus subtilis*).
2. Create gradients of known cell densities (10²–10⁵ cells/particle).
3. Process individual particles using the full single-particle omics workflow (DNA extraction, WGA, sequencing).
4. Compare observed read distributions, genome coverage, and ARG/virulence gene recovery to expected values.

**Expected Output:**
Correlation between input cell abundance and sequencing-derived microbial load.

**Validation Metric:**
Regression slope ≈ 1 (±10%) between expected vs. measured abundance; false-positive rate from blanks < 1%.
Used to calibrate the microbial load threshold (Tₘ) for defining colonized status.

**3. Bulk–Particle Comparative Validation**

**Objective:**
Quantify how bulk ‘omics’ signals misrepresent true prevalence and identify the degree of loud-minority bias.

**Rationale:**
Bulk sequencing conflates presence with prevalence. Comparing bulk to particle-resolved outputs on the same material defines how strongly a few supercarriers dominate aggregate signals.

**Experimental Steps:**

1. Collect an environmental sample (e.g., surface water, beach sediment) and divide it into two identical subsamples.
2. Process one via standard bulk ‘omics’ (pooled extraction, sequencing) and the other via particle-resolved workflow (≥200 particles).
3. Compute bulk-normalized microbial load and ARG abundance.
4. Derive CPI and colonization-intensity histogram from the particle-resolved dataset.
5. Estimate what proportion of total bulk signal is contributed by the top 5–10% of particles.

**Expected Output:**
Distribution of microbial load showing right-skew and quantification of “loud minority” effect.

**Validation Metric:**
Supercarrier dominance index (SDI) = % of total reads or biomass from top 10% of particles.
SDI > 50% confirms skewed distribution and validates need for particle resolution.

**4. Temporal Persistence and Supercarrier Stability**

**Objective:**
Assess whether supercarrier states are transient or persistent over time.

**Rationale:**
Understanding colonization dynamics is crucial for defining long-term vector potential.

**Experimental Steps:**

1. Deploy known particles (pristine and weathered PE, PP, PET fragments) in situ using mesh enclosures across different habitats (marine, estuarine, freshwater).
2. Retrieve replicate subsets at defined intervals (e.g., 1 week, 1 month, 3 months, 6 months).
3. Process each timepoint using imaging (biofilm coverage) and omics (taxonomic and functional profiling).
4. Calculate CPI and identify whether the same particles (tagged by shape/ID) maintain or lose heavy colonization over time.

**Expected Output:**
Temporal CPI trajectories and persistence patterns of supercarriers.

**Validation Metric:**
Supercarrier persistence rate (SPR) = proportion of initially colonized particles remaining heavily colonized after t months.
SPR > 0.5 suggests ecological stability; rapid turnover (<0.2) implies transient colonization.

**Integration and Interpretation**

Results from these validation modules provide the quantitative foundation for CPI calibration:

- **Spike-Recovery →** extraction/imaging correction factor
- **Mock-Community →** sequencing detection thresholds and blank correction
- **Bulk–Particle Comparison →** loud-minority bias quantification
- **Temporal Persistence →** ecological longevity of vector states

Together, they establish traceability between observed CPI values and underlying ecological reality, converting the framework from a conceptual advance into a verifiable analytical standard.

**Text S2 | Worked Example of CPI Calculation and Interpretation**

**Purpose:** This supplementary text provides a step-by-step, hypothetical illustration of how the Colonization Prevalence Index (CPI) is calculated and interpreted from single-particle imaging data. This example is designed to demonstrate the practical workflow of the proposed particle-resolved framework and to clarify the transformation of raw particle-level measurements into an ecologically meaningful population metric.

**Scenario:** A study isolates 150 environmental microplastic particles (50–500 µm) from freshwater sediment. Each particle is analyzed via epifluorescence microscopy following staining with SYBR Gold (nucleic acids) and Concanavalin A conjugated to a fluorophore (extracellular polysaccharides). These stains provide a combined signal representing total microbial biofilm biomass and matrix.

**Step 1: Image Analysis and Data Generation**

Automated image segmentation (using software such as CellProfiler or a U-Net-based model) is performed on each particle image. The output for each particle is a single value: the **percentage of its surface area covered by fluorescent signal** (*Biofilm Coverage*).

**Step 2: Determining the Colonization Threshold (*Tb*)**

A subset of 30 particles is manually curated by an expert to establish ground truth ("colonized" vs. "not colonized"). Using this validation set, a Receiver Operating Characteristic (ROC) curve is generated by testing a range of potential coverage thresholds (e.g., from 5% to 50%). The **Youden Index (J = Sensitivity + Specificity – 1)** is calculated for each threshold. The threshold that maximizes J is selected as the objective, study-specific *Tb*. For this hypothetical dataset, the optimal *Tb* is **15% surface coverage**.

**Step 3: Calculating the CPI**

The CPI is calculated as the proportion of particles in the full dataset whose biofilm coverage exceeds *Tb*.

- **Nc (Number of colonized particles):** 36 particles have coverage > 15%.
- **Nt (Total particles analyzed):** 150.
- **CPI:** (Nc / Nt) × 100 = (36 / 150) × 100 = **24.0%**.

**Step 4: Estimating Statistical Uncertainty**

To account for sampling variability, a **non-parametric bootstrap** procedure is performed:

1. 10,000 random resamples (with replacement) of size 150 are drawn from the original dataset.
2. The CPI is recalculated for each resample.
3. The 2.5th and 97.5th percentiles of the resulting 10,000 CPI values define the 95% confidence interval (CI).

For this dataset, the 95% CI is **18.0% to 32.0%**.

**Step 5: Interpreting the Result**

- **Metric Value:** A CPI of **24.0%** indicates that under the defined conditions (habitat, particle size, threshold), approximately one-quarter of the microplastic population was substantially colonized.
- **Risk Context:** Following the proposed interpretive framework (Section 6), this falls within the **Low-Moderate** colonization prevalence category (CPI = 10–50%).
- **Ecological Implication:** The result directly challenges the assumption of universal colonization. It demonstrates that vector potential is a property of a distinct particle sub-population. The majority of particles (76%) were lightly colonized or bare, suggesting that ecological and exposure risks are likely concentrated rather than diffuse.
- **Confidence & Reporting:** The finding should be reported as: **CPI = 24.0% (95% CI: 18.0–32.0%)**. The CI reflects the precision of the estimate given the sample size; a narrower CI could be achieved by analyzing more particles.

**Conclusion for Pathway A:** This worked example demonstrates how particle-resolved imaging data is processed to yield a statistically robust, interpretable measure of colonization prevalence. The same logical workflow applies to Pathway B (omics), where the threshold (*Tm*) would be derived from negative control distributions and the "signal" would be microbial load (e.g., 16S rRNA gene copies).

Once the foundational CPI is established, researchers may optionally calculate a Weighted CPI (CPIw) to incorporate colonization intensity. Using the same biofilm coverage data (Ii), CPIw can be derived as (1/Nt) Σ (Ii / Imax), where Imax is the maximum coverage observed. This provides a continuous metric that gives greater weight to heavily colonized 'supercarrier' particles.

**Text S3 | Minimum Reporting Checklist for Particle-Resolved Ecology and CPI Studies**

To ensure reproducibility and comparability of the Colonization Prevalence Index (CPI) and associated metrics, the following metadata and methodological parameters should be reported for each dataset or study site. These fields define the minimum reporting standard for future particle-resolved plastisphere research.

**A. Sampling and Environmental Context**

- **Sampling date and location:** Include geographic coordinates (GPS), habitat type (marine surface, sediment, soil, freshwater, biota), and depth or altitude where relevant.
- **Environmental parameters:** Temperature, salinity, pH, nutrient load, and organic matter content.
- **Matrix description:** Water, sediment, soil, tissue, or biotic material type.
- **Sampling effort:** Total sample volume or mass processed (e.g., 10 L water, 200 g sediment).

**B. Extraction and Recovery**

- **Bulk extraction protocol:** Specify density-separation medium, mesh size, and digestion/cleaning steps (e.g., NaI separation, enzymatic digestion, peroxide treatment).
- **Recovery efficiency estimate:** Provide calculated recovery (% of spiked or visually confirmed particles recovered).
- **Size fractionation:** Define particle size bins analyzed (e.g., 20–50 µm, 50–500 µm).
- **Selection criteria:** Indicate whether particles were hand-picked, randomly subsampled, or size-class standardized.

**C. Imaging and Morphological Profiling (Pathway A)**

- **Microscope type and model:** e.g., confocal, epifluorescence, or flow-imaging cytometer.
- **Fluorescent stains or probes:** Include dye names, excitation/emission wavelengths, and concentrations.
- **Image acquisition parameters:** Pixel resolution, magnification, z-stack spacing, and field-of-view dimensions.
- **Segmentation algorithm:** Specify image analysis software, machine-learning model (if used), and version number.
- **Threshold for colonization (T_b):** Define coverage cutoff (% surface area colonized) and method for empirical derivation (e.g., background + 2σ noise).

**D. Molecular Profiling (Pathway B)**

- **DNA/RNA extraction method:** Specify kit or custom protocol, input biomass, and particle handling precautions.
- **Amplification protocol:** Include WGA (if used), primer sets, and cycling conditions.
- **Sequencing platform and parameters:** Platform type (Illumina, Oxford Nanopore, PacBio), read length, and depth.
- **Negative and procedural blanks:** Number processed, contamination detection method, and background correction applied.
- **Threshold for colonization (T_m):** Define microbial load or read-count cutoff for “colonized” classification, and provide justification (e.g., >75th percentile of local background).

**E. CPI Calculation and Statistical Analysis**

- **CPI and weighted CPI formulas:** Specify exact form used, referencing main text equation.
- **Sample size (N_t):** Number of individual particles analyzed per habitat or treatment.
- **Confidence interval estimation:** Specify whether bootstrapping, binomial, or Bayesian methods were used.
- **Statistical analyses:** Describe correlation or regression models linking colonization to particle traits (e.g., polymer type, roughness).
- **Distributional data:** Include raw or binned colonization intensities for transparency.

**F. Data Accessibility**

- **Raw imaging data:** Link to image repository (e.g., Zenodo, Dryad, or institutional server).
- **Sequence data:** Accession numbers for metagenomic or amplicon datasets (NCBI SRA, ENA).
- **Metadata table:** CSV or Excel file with per-particle measurements (polymer type, size, colonization intensity, microbial load).
